# Improving Metabolic and Cardiovascular Health at an Early Psychosis Intervention Program in Vancouver, Canada

**DOI:** 10.3389/fpsyt.2014.00105

**Published:** 2014-09-05

**Authors:** Diane H. Fredrikson, Heidi N. Boyda, Lurdes Tse, Zachary Whitney, Mark A. Pattison, Fred J. Ott, Laura Hansen, Alasdair M. Barr

**Affiliations:** ^1^Faculty of Medicine, University of British Columbia, Vancouver, BC, Canada; ^2^Early Psychosis Intervention Program, Vancouver Coastal Health, Vancouver, BC, Canada

**Keywords:** psychosis, antipsychotic, metabolic syndrome and diabetes mellitus, exercise, health coaching, mental illness, schizophrenia

## Abstract

Psychotic disorders most commonly appear during the late teenage years and early adulthood. A focused and rapid clinical response by an integrated health team can help to improve the quality of life of the patient, leading to a better long-term prognosis. The Vancouver Coastal Health early psychosis intervention program covers a catchment area of approximately 800,000 people in the cities of Vancouver and Richmond, Canada. The program provides a multidisciplinary approach to supporting patients under the age of 30 who have recently experienced first-break psychosis. The program addresses the needs of the treatment environment, medication, and psychological therapies. A critical part of this support includes a program to specifically improve patients’ physical health. Physical health needs are addressed through a two-pronged, parallel approach. Patients receive routine metabolic health assessments during their first year in the program, where standard metabolic parameters are recorded. Based on the results of clinical interviews and laboratory tests, specific actionable interventions are recommended. The second key strategy is a program that promotes healthy lifestyle goal development. Patients work closely with occupational therapists to develop goals to improve cardiometabolic health. These programs are supported by an active research environment, where patients are able to engage in studies with a focus on improving their physical health. These studies include a longitudinal evaluation of the effects of integrated health coaching on maintaining cardiometabolic health in patients recently admitted to the program, as well as a clinical study that evaluates the effects of low versus higher metabolic risk antipsychotic drugs on central adiposity. An additional pharmacogenomic study is helping to identify genetic variants that may predict cardiometabolic changes following treatment with antipsychotic drugs.

## Introduction

The age of onset of schizophrenia spectrum disorders and most other forms of idiopathic psychosis is typically during the later teenage years through the early- to mid-20s. There is usually a period of cognitive and functional decline prior to the first psychotic break, which may be referred to as the “prodrome” (1). This prodrome is characterized by changes from premorbid functioning that may be expressed in ways such as social withdrawal, loss of employment, and decreased interest in academic or vocational activities (2). The subsequent period associated with the initial onset of overt psychotic symptoms represents a traumatic period not only to the individual affected but also for family and friends (3). This stress may be exacerbated by the lack of awareness among the general population of the biological nature of psychotic disorders, as well as the highly variable presentation of early psychotic symptoms and the stigma associated with mental illness in general.

In recent years, there has been an increased awareness of the need for programs that provide focused support specifically for patients with a recently diagnosed psychotic disorder. The rationale for such “early psychosis intervention (EPI)” programs is based in significant part on the biological hypothesis that during the early stages of psychotic disorders, the brain’s plasticity remains largely intact. Hence, optimal treatment with antipsychotic medications and psychotherapy represents the best opportunity to retain long-term function in what will be essentially a life-long disorder for many individuals (4). The earlier stages of psychosis are also associated with the greatest risk of numerous complications, such as social isolation, development of drug use and criminality, and suicide (5). Importantly, patient and family attitudes toward treatment may be most receptive in the early phases of the illness (6). As a whole, research on the effectiveness of EPI studies has indicated the key value of such programs, demonstrating that patients whose psychosis remains untreated for longer periods of time have a significantly poorer long-term outcome, reflected in worse functional performance, lower rates of remission, and decreased quality of life (7). EPI programs have also been demonstrated to lead to better clinical and functional outcomes than standard care programs for extended periods of time (8–10), even after individuals have left the program.

### The Vancouver/Richmond early psychosis intervention program

The Vancouver/Richmond EPI program provides specialized support for patients and their families, who have recently experienced issues with psychosis. The program covers a catchment area for the cities of Vancouver and Richmond (combined population: 793,000) in the southwestern region of the province of British Columbia, Canada, and is supported publically through the Vancouver Coastal Health Authority – a government agency. The program accepts patients between the ages of 13 and 30 with recent onset symptoms of psychosis. Team members at the program include psychiatrists (including those with specialized experience in treating children and adolescents), psychiatric nurses, social workers, child and family therapists, occupational therapists, and case workers. The services that are available at the program include individualized care in the following areas:

- Assessment
- Treatment
- Medication management
- Second opinions
- Consultations
- Case management
- Support and recovery
- Prevention of relapse
- Support returning to work or school
- Assistance to families to manage their family life in relation to their family member
- Patient and family psychoeducation.

Referrals to the Vancouver/Richmond EPI program come from a widespread number of different sources. These include the regional health authority central intake lines, hospitals, primary care physicians, secondary and post-secondary schools, and family members or potential patients who have heard about the program through community sources. A number of additional services have been developed, which focus on maintaining the metabolic and cardiovascular health of the patients (these are described in more detail below). Furthermore, a number of ongoing research studies at the program with a focus on the biological basis of metabolic dysregulation in patients treated with antipsychotic drugs provide additional opportunities for patients to learn about their physical health.

### Cardiometabolic health in patients treated with antipsychotic drugs

It is now well known that individuals with a psychotic disorder who are treated with an antipsychotic drug are significantly more likely to exhibit poor metabolic and cardiovascular health compared to the general population (11). Life expectancy may be reduced by up to 25 years in patients with schizophrenia, which predominantly reflects increased morbidity and mortality from causes other than suicide, namely, type 2 diabetes mellitus (DM) and cardiovascular disease (12). The factors that contribute to poor health are those associated with an unhealthy lifestyle, including a non-nutritious diet, lack of exercise, and high rates of smoking (13–15). However, an additional factor that has been the focus of considerable research over the past decade is the direct cardiometabolic effects of the antipsychotic drugs themselves (16–19). Antipsychotic drugs can be given for a wide range of different psychiatric indications, in both adults and youth (20–23).

The second generation antipsychotic drugs (atypicals), in particular, have been associated with severe metabolic dysregulation. These effects are so severe that the American Diabetes Association, the American Psychiatric Association, the American Association of Clinical Endocrinologists, and the North American Association for the Study of Obesity co-sponsored a Joint Consensus Statement, providing guidelines for assessment of metabolic risk for all patients being treated with atypical antipsychotic drugs (24).

Weight gain is the most commonly reported adverse effect with the atypical antipsychotics, likely due to its prominence and ease of measurement. Based on the National Cholesterol Education Programs Adult Treatment Panel III, clinical definition of the metabolic syndrome requires the presence of at least three risk factors (25). Among patients with metabolic syndrome, the relative risk for type 2 DM and coronary heart disease is 1.5–5 times that of the general population (26). Numerous studies have reported high rates of metabolic syndrome in atypical treated patients, with prevalence rates of over 50% for prediabetes or type 2 DM in some adult psychiatric inpatient settings (27–29). In the large head-to-head clinical trial of atypical antipsychotics, the Clinical Antipsychotic Trial of Intervention Effectiveness (CATIE) Study (a major, multi-center trial sponsored by NIMH) observed that 43% of patients treated with atypicals had metabolic syndrome. Controlling for BMI, CATIE men were 85%, and CATIE women 137% more likely to have metabolic syndrome than non-psychiatric subjects (30). Importantly, evidence indicates that youth appear to be at higher risk than adults for antipsychotic-induced weight gain and associated metabolic abnormalities (31–33), emphasizing the need for close monitoring of patients in EPI programs.

## Programs to Improve Cardiometabolic Health

A number of different programs have been implemented at the Vancouver/Richmond EPI program with the specific goal of improving the physical health of patients who are treated by the multidisciplinary team at the site. Patients are made aware of these programs as soon as their initial meeting with a psychiatrist. All programs are completely voluntary and free of charge, although all patients are strongly encouraged to take part in these optional activities. Patients’ families are also provided with information about these programs to help enlist their support in encouraging patients to participate.

In these programs, patients routinely meet in a group format to discuss strategies to learn about and improve their physical health. When health-related goals are established, plans are structured according to the SMART criteria (i.e., goals must be *S*pecific, *M*easurable, *A*chievable, *R*ealistic, and *T*ime related) (34). Whenever possible, goals are set as precisely as possible. In setting a precise goal, including dates, times, and amounts, it allows progress to be measured and thus the patient has a clearer sense of whether the exact goal has been achieved or they are at least making advances. When there are several goals involved, the patient is encouraged to set priorities. This can prevent the patient from feeling overwhelmed by having too many goals, and draws attention toward priorities. When setting goals, writing them down can avoid confusion and lend them more force. It is also important to keep operational goals small; if a goal is too large, progress can appear difficult. Keeping goals modest and incremental provides more opportunity for reward.

When initially setting goals, patients are encouraged to think a goal through, with regard to the following questions:

- What skills do I need to achieve this?
- What information or knowledge do I need?
- What help, assistance, or collaboration do I need?
- What resources do I need?
- What can block my progress?
- Am I making any assumptions?
- Is there a better way of doing things?

After a goal has been set by the patient, they will complete a Healthy Lifestyle Goal Development Worksheet. Although completed individually, these worksheets are reviewed as a group, and a photocopy is placed in the patient’s medical chart.

### Metabolic monitoring program

A key protocol that is supported by the Vancouver/Richmond EPI program is the Metabolic Monitoring Program. As noted above, it is now recommended that all patients treated with atypical antipsychotic medications be screened routinely for metabolic abnormalities after starting treatment (24). For a number of different reasons, including traditional boundary issues, psychiatrists usually do not take direct anthropometric measurements of their patients during clinical appointments. In an outpatient setting, which represents the context of the typical EPI program (35), patients will be seen separately by their primary care provider for issues of general health, which may include cardiometabolic side effects related to medications. However, this can create a disconnection between the primary health care provider and the patient’s psychiatrist, and result in less thorough or frequent metabolic screening (36, 37).

At the Vancouver/Richmond EPI program, a medical specialist trained in medical biochemistry with extensive experience in treating endocrine issues related to cardiometabolic disorders runs a clinic once per month. At that time, patients in the program are encouraged to follow an established screening algorithm, which monitors patients’ cardiometabolic health closely through the first two years in the program. According to this algorithm, each patient should be screened at baseline entry into the psychosis program and 1, 2, 3, 6, 9, and 12 months, and then once per year afterward. During the metabolic workup, the specialist obtains a comprehensive overview of the cardiometabolic health of the patient, enabling an accurate estimation of overall health risk factors. Indices measured include height and weight (BMI), waist circumference (while ethnicity is also noted to account for differences in risk based on ethnic background norms), resting blood pressure, resting heart rate, fasting plasma glucose, fasting total cholesterol, fasting LDL, fasting HDL, fasting triglycerides, and fasting insulin levels for patients under 17 years old. These values are reviewed to determine if criteria have been met for metabolic syndrome, and then considered in combination with other risk factors such as smoking status and current medications to determine the overall health risk, and whether specific interventions may be necessary (38, 39).

Interventions available include education of the patient about the nature of metabolic risk for patients with psychosis who are treated with antipsychotic drugs. The signs and symptoms of type 2 DM and diabetic ketoacidosis may be reviewed explicitly, and lifestyle management options are discussed in depth. Self-management goals will be developed, where the patient is encouraged to reduce or quit smoking, improve eating habits, increase physical activity, decrease the amount of time spent each day sitting, and also improve stress management and sleep habits. Finally, the medical specialist may prescribe medications to improve metabolic health or provide a referral to another medical specialist or general practitioner. Referrals are also made to smoking cessation support programs, exercise programs, and dieticians.

## Health-Related Research at the Vancouver/Richmond Early Psychosis Intervention Program

As noted above, patients who have recently experienced the onset of a psychotic disorder and begun treatment with antipsychotic medications are at significantly increased risk of worsened cardiometabolic health, and ultimately developing disorders such as type 2 DM and cardiovascular disease, leading to premature death. In most cases, the biological nature of the metabolic dysregulation observed in patients treated with antipsychotic medications is not well understood, as the mechanism by which antipsychotic drugs cause glucose intolerance, insulin resistance, weight gain, and hyperlipidemia remains under investigation (40). It is particularly important to study cardiometabolic changes in patients who have recently experienced the full onset of their psychotic disorder and begun drug treatment for several reasons. First, this may be the period of most rapid physical change, particularly as patients are starting from a healthier baseline and some may have not yet reached full sexual maturity (33). Second, patients have less of a “history” with previous antipsychotic medications, and are less likely to be treated with multiple antipsychotic drugs or additional classes of psychotherapeutic medications (20), meaning that observed physical changes can be interpreted with greater clarity. Finally, the greater receptivity of patients and family during this phase of the illness (6) is conducive to engaging the patients in the type of longitudinal research studies that will ultimately be necessary for progress to be made in understanding these issues.

At the Vancouver/Richmond EPI program, there are currently multiple active research studies, which focus on understanding – and ultimately improving – the cardiometabolic health of patients with a psychotic disorder. In most cases, these studies represent direct translational research from our basic laboratory studies, which currently focus on understanding the biology (41–45) and treatment (46–48) of antipsychotic drug-induced metabolic dysregulation. We briefly discuss several of these ongoing studies below. All studies are conducted with full approval by the ethical review board of the University of British Columbia and in accordance with national guidelines for clinical trials, and have been registered with www.clinicaltrials.gov.

### Is health coaching effective for improving metabolic health in people with psychotic disorders? (clinicaltrials.gov identifier NCT01752465)

#### Background

Integrative Health Coaching is a term used to describe an inclusive approach to improve the health of patients at the individual level. It requires the application of evidence-based health psychology and coaching principles to assist clients to achieve positive health outcomes through cognitive and behavior change. Health Coaching is typically conducted in the context of disease prevention and/or chronic condition self-management within public and private sector health services. It is suitable for interdisciplinary teams of health professionals within chronic disease prevention, early intervention, rehabilitation, and other chronic condition self-management programs. Studies have confirmed the value of this approach, such as the Duke Integrative Medicine Study, which began in 2002 with a study funded by the Centers for Medicare and Medicaid. This randomized clinical trial found that health coaching intervention significantly reduced the 10-year prospective risk of coronary heart disease (49). Additionally, other research studies have reported that health coaching can lead to greater weight loss in subjects following pregnancy (50) and in subjects with glucose intolerance (51) or Type 2 DM (52). The benefits of this type of program in patients who have recently been diagnosed with a psychotic disorder for the first time, such as schizophrenia, remain unknown.

#### Hypothesis

Based on the proven value of Integrative Health Coaching Programs in improving the long-term health of patient populations with regard to obesity, type 2 DM, and cardiovascular risk, we are testing the following hypothesis: in a first-episode psychosis population that has recently begun treatment with atypical antipsychotic drugs, the inclusion of an Integrative Health Coaching goal-setting model will significantly improve metabolic health outcomes compared to usual treatment.

#### Protocol

We are conducting a non-blinded clinical trial of the efficacy of Integrative Health Coaching techniques in 40 subjects who are currently treated at the Vancouver/Richmond EPI program. All subjects are randomly assigned to one of two treatment groups (*n* = 20 subjects per group). In the first group, subjects receive treatment for psychosis based on the current standard of care, which includes psychosocial training (social skills training, cognitive therapy, stress reduction training, and vocational rehabilitation), education, and pharmacological treatment. The second group of subjects receives not only the standard of care treatment for psychosis but also additional Integrative Health Coaching to improve physical health (see below). For inclusion in the study, subjects must be prescribed an atypical antipsychotic drug. Intake into the study is naturalistic, as the subject’s psychiatrist at the early psychosis program is free to prescribe whatever psychotropic medication they decide is best for the subject. Subjects in both treatment groups are given assessments of physical health, with a particular emphasis on metabolic dysregulation, at months 1, 2, 3, and 6 from when they enter the study. Subjects are characterized with clinical data on demographics, health history, prior and concomitant medications, and metabolic indices (e.g., height, weight, waist circumference, resting blood pressure and heart rate, fasting plasma glucose, total cholesterol, LDL-C, HDL-C, triglycerides, insulin, etc.). This information is collected via chart review. Diagnoses are abstracted from reports and notes written by physicians and case managers at Vancouver Richmond EPI. This includes the clinical diagnoses at intake and, if applicable, any changes in clinical diagnosis at the time of discharge, as well as descriptions of positive and negative symptoms, abnormalities in affect, problems in social and occupational functioning, and any cognitive problems. Data are collected and analyzed for differences between the two groups at the end of the study. Results will be analyzed based on intent-to-treat analysis, and comparison of metabolic and other health indices compared between the two groups using t-test and chi-square analyses. Based on prior studies using Integrative Health Coaching techniques and the reported effect sizes, we determined that the present study with 40 subjects will have 80% power to detect a true treatment difference in the change of metabolic improvement of 0.5 SD. For example, behavioral health coaching in patients with type 2 DM has been shown to exert effect sizes as large as Cohen’s *d* = 1.50 on behavioral measures, although physiological changes may be lower (53).

Self-management techniques are utilized to facilitate patient engagement in their own health care, including participating in lifestyle activities that protect and promote physical health, learning to monitor and manage symptoms and signs of metabolic dysregulation, and learning to manage the impact of metabolic side effects on functioning, while strongly promoting adherence to current treatment regimens. Training identifies the patient as an expert in their own health recovery and supports the individual to find their own solutions to problems, based on the use of intrinsic motivators. Subjects identify specific, individualized goals with positive imagery, and are trained to overcome barriers to change. For example, rather than simply state “I want to lose 10 kg,” the subject will be coached to develop specific goals and action plans, such as committing to walking three times per week for 30 min on Mon–Wed–Fri and replacing take-away foods for dinner with healthy home cooked meals three times per week. Subjects receive 1 h of Health Coaching twice monthly during the first 3 months of the study, and then monthly for the remaining 3 months.

#### Outcome measures

We use routine measures of metabolic health, which are used for all patients who enter the EPI program. We record all of the indices that are required to make an evaluation of metabolic syndrome. It includes information on obesity (BMI, waist circumference), blood pressure, fasting plasma glucose, fasting cholesterol (LDL and HDL), and fasting triglycerides. We also measure liver function (AST and ALT) as well as other relevant metabolic parameters. We also ask subjects to complete self-report scales of the Short Form-36 instrument, which is a well-established tool to measure health and well-being in psychiatric patients (54). Subjects also complete the Three-Factor Eating Questionnaire, which is a validated tool comprising 51 items that measure three components of eating behavior (55). Factor I reflects conscious mechanisms for limiting food intake, factor II reflects a variety of disinhibition behaviors, while factor III reflects feelings of hunger and its behavioral consequences. Psychotropic medication prescription information is obtained from medical charts, while each subject is asked about their medication adherence using a standardized questionnaire.

#### Anticipated results

We predict that subjects who only receive the standard of care treatment will exhibit worse metabolic outcome measures over the duration of the 6 months, compared to baseline, as the metabolic effects of antipsychotic medications become greater over time. In contrast, we predict that metabolic indices will show significantly less development in subjects receiving Health Coaching. This latter group will also show better general health, well-being, better eating behaviors, and stronger medication adherence. If our hypothesis is proven to be correct, then we will have demonstrated for the first time in a population with psychosis that Integrative Health Coaching can significantly improve metabolic and general health. This will improve morbidity, long-term mortality, and quality of life in patients, and may represent a considerable cost savings to the health care system using a set of techniques that are relatively inexpensive and easy to implement.

### A comparison of aripiprazole versus higher metabolic risk antipsychotic drugs on adiposity using MRI (CALM) (clinicaltrials.gov identifier NCT01739127)

#### Background

Evidence indicates that patients who have just started treatment with antipsychotic drugs are at higher risk for antipsychotic-induced weight gain and associated metabolic abnormalities than older patients (31). A recent meta-analysis indicated that switching from other atypical antipsychotics to the antipsychotic drug aripiprazole resulted in significant weight loss and may be an optimal treatment for patients who exhibit drug-induced weight gain (56). Weight gain is a common adverse effect with most of the atypical and some of the typical antipsychotic drugs. Tandon and Halbreich reviewed the relative propensity of all atypical antipsychotic drugs to induce weight gain in adults (57), and expressed the order as follows: clozapine > olanzapine > risperidone = quetiapine > ziprasidone = aripiprazole. It is important, though, to note that weight gain *per se* is not necessarily a strong predictor of subsequent metabolic and cardiovascular complications. Evidence from the extensive diabetes literature indicates that there are major differences between intra-abdominal visceral fat (defined as fat accumulation around the viscera and intra-abdominal solid organs) versus peripheral/subcutaneous fat in the pathogenesis of metabolic syndrome. Visceral fat is a highly active endocrine organ, and levels of visceral fat are a much stronger predictor of mortality than other types of body fat (58). The overwhelming majority of studies have demonstrated that when compared directly, levels of visceral fat are a much better predictor of insulin resistance and hyperglycemia (two of the core symptoms of type 2 DM) than levels of subcutaneous fat (59, 60). For example, Usui and colleagues reported in 2010 that levels of visceral fat were four times more accurate than levels of subcutaneous fat in predicting insulin resistance (61). Despite their widespread use, anthropometric measures of adiposity, such as BMI and waist circumference, have been subject to criticism by experts (62). Importantly, several recent studies have reported that intrahepatic lipid levels are at least as accurate a predictor of glucose intolerance as levels of visceral fat (63–65). Hepatic steatosis, or “fatty liver,” is a well-known pathological marker of numerous metabolic disorders.

While visceral fat and intrahepatic lipids are, therefore, a more accurate predictor of metabolic dysregulation and potential cardiovascular complications, these indices are not readily acquired. Indirect measures of adiposity, such as measurement of waist circumference and BMI, are cheap and convenient techniques, but they do not provide accurate quantitative assessment of abdominal visceral fat, or regional distribution in and around specific organs, such as the liver. This is a critical shortcoming, for reasons described above, as well as because there are major gender differences in the accumulation and amount of visceral fat that cannot be controlled for based on peripheral measures.

The “gold standard” technique of fat measurement, therefore, has been magnetic resonance imaging (MRI), which enables both the amount and regional distribution of visceral fat to be determined non-invasively. This technique is considerably more accurate than dual X-ray absorptiometry (DEXA) and does not expose subjects to the potentially harmful effects of radiation associated with DEXA or CT scans. To date, there has been only one study that reported the effects of antipsychotic drugs on visceral fat deposition using MRI (66). This study, in Chinese Han adults, showed a marked increase in visceral fat – which was significantly greater than changes in subcutaneous fat – after 10 weeks of treatment with either risperidone or chlorpromazine, assessed using a low-field-strength scanner (0.2 T). There has never been a study of the effects of antipsychotic drugs on intrahepatic lipid levels, which is assessed using magnetic resonance spectroscopy (MRS). The effects not only of aripiprazole but also all other antipsychotics on visceral fat accumulation and intrahepatic lipid levels in adolescents remain completely unknown. The goal of the current study is to study aripiprazole in the first ever study of antipsychotic-induced weight gain on levels and regional distribution of visceral versus subcutaneous fat in EPI patients using MRI.

#### Hypotheses

1. Patients treated with a lower metabolic risk antipsychotic drug (aripiprazole) will exhibit no significant difference in metabolic indices, including MRI/MRS measures, compared to control subjects at either the beginning of the study or after 16 weeks of drug treatment.
2. By contrast, patients treated with antipsychotic drugs that represent a higher metabolic risk (risperidone, quetiapine, or olanzapine) will exhibit significant metabolic changes, such as increased visceral fat and greater lipid infiltration of the liver, compared to vehicle and aripiprazole-treated patients. This effect will be modest at the time of the initial scan, and substantially greater at the time of the second scan.
3. The significant increase in the amount of visceral fat and lipid infiltration of the liver following antipsychotic drug treatment with the higher risk drugs will correlate strongly with metabolic dysregulation, such as higher fasting glucose levels and insulin resistance.

#### Protocol

*Design:* The study is a longitudinal, naturalistic intake study. We are recruiting 90 subjects, who are interviewed and receive MRI scans on entry into the study and again after 16 weeks. Of these, 30 are age- and gender-matched healthy subjects who serve as controls. For the remaining 60 subjects treated with antipsychotic drugs, there are two treatment groups, based on the metabolic liability of the antipsychotic drugs they are treated with. We are recruiting 30 subjects treated with aripiprazole, which represents the low-risk group, and 30 subjects treated with higher metabolic risk drugs (risperidone, quetiapine, and olanzapine). Groups are matched on sex and age. At entry into the study, subjects receive an abdominal MRI/MRS scan to measure fat and lipid composition of the liver. Metabolic tests to complement the fat imaging are also performed to gather an independent index of glucose intolerance and insulin resistance.

Subjects must have received no more than 6 months lifetime total of prior treatment with antipsychotic drugs. Anthropometric measures, which include height, weight, and waist circumferences, as well as laboratory evaluations performed in the fasting state, are obtained. To assess glucose intolerance, we conduct an oral glucose tolerance test (1.75 g/kg of glucola with maximum dose of 75 g administered following a 12 h fast). Height, weight, blood pressure, pulse, and waist circumference are obtained on the day of the oral glucose tolerance test. Fasting lipid profile (total cholesterol, triglycerides, LDL-C, HDL-C), leptin, adiponectin, and proinsulin are also measured. Antipsychotic drug levels are measured by HPLC to ensure medication adherence.

Clinical measures include:

- Positive and negative syndromes scale (PANSS)
- Calgary depression scale (CDS)
- Mini-international neuropsychiatric interview (MINI v6.0)
- Social and occupational functioning scale (SOFAS)
- Anthropomorphic assessments for basal metabolic index (height, weight, BMI, blood pressure, pulse, waist circumference).

**Figure 1:**
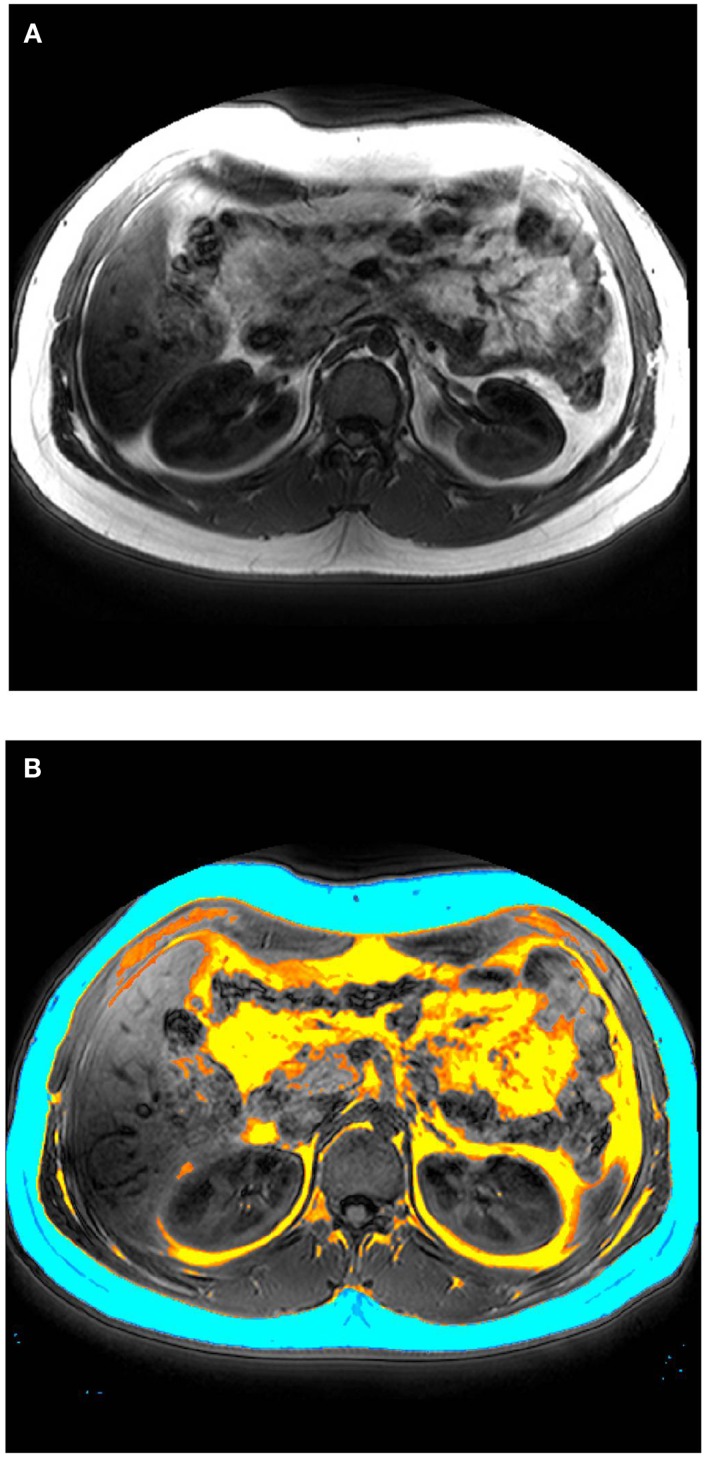
**(A)** Representative MRI image (axial plane) performed on a 3-T clinical scanner. **(B)** Total visceral fat (yellow), subcutaneous fat (blue), and intramuscular fat (orange) manually segmented.

After laboratory and cognitive tests have been completed, subjects complete an abdominal scan with the MRI scanner at UBC Hospital. Image acquisition are performed on a 3-T clinical MRI scanner (Philips System), using a body coil and a T1 weighted acquisition of 200 contiguous 1 mm axial slices of the abdomen, between the lower rib margin and the iliac crest. Scan time is 45 min (plus approximately 10 min extra time in the scanner for alignment). Total visceral fat, subcutaneous fat, and intramuscular fat are segmented automatically (see Figure 1); regional distribution of fat is determined by manual segmentation. MRS quantifies lipid peaks to measure lipid content of a liver-based voxel (scan time = 15 min). All subjects are followed up 16 weeks later for a second interview and MRI/MRS scan. Drug adherence is assessed using techniques that we have used previously. Data are analyzed using an “intent-to-treat” statistical approach. In addition to the main intent-to-treat analysis, we will conduct a secondary “completers” analysis with only subjects who did not switch from one class of antipsychotic drugs to the other. Group sizes are sufficiently powered to detect significant differences, based on power calculations and our prior observations. Linear regression analysis will be performed to determine the relationship between visceral or subcutaneous fat and liver lipids versus metabolic changes.

#### Anticipated significance

The present study will be the first ever to use MRI to assess the effects of atypical antipsychotic drugs on levels of visceral and subcutaneous fat, as well as lipid infiltration of the liver, in patients recently starting antipsychotic drugs. It will also be unique in that it will employ these procedures to compare the metabolic side effects of atypical drugs with low versus higher metabolic side effect risks. The results of this study could provide important information about the potential hidden harmful effects associated with atypical drug use for atypicals, as there is little knowledge about the effects of these pharmacotherapies on visceral fat accumulation in patients. The data accumulated may ultimately be useful for the development of treatment guidelines. Clear evidence of increased adiposity in higher metabolic risk antipsychotic drug-treated patients could provide strong impetus for prescribers to use low metabolic risk drugs to improve cardiometabolic health in patients with psychosis.

### Adverse events and genomics in schizophrenia (AEGIS) (clinicaltrials.gov identifier NCT01966588)

#### Background

As noted above, many of the atypical antipsychotic drugs commonly cause serious metabolic disturbances, including weight gain, hyperlipidemia, glucose intolerance, and insulin resistance, resulting in higher rates of cardiometabolic disorders. Extensive research has shown that specific antipsychotic drugs or combinations thereof may provide a differential risk for metabolic dysregulation in patients. There are large inter-individual differences in vulnerability to antipsychotic drug-induced side effects. An emerging body of evidence in the field of pharmacogenomics indicates that these inter-individual differences may be due in significant part to genetic factors, either as inherited or *de novo* genetic variation (67, 68). However, the key genes or biological targets involved remain largely unknown, due to the complexity of the clinical picture, where diverse factors such as activity levels, hunger, diet, substance use, concurrent medications, and choice of antipsychotic drug can all influence the risk for metabolic dysregulation. Furthermore, few comprehensive genetic studies have addressed this question, and those that have been performed have either used a candidate gene approach or have assessed only common variants. Comprehensive, high-resolution pharmacogenomic studies are challenging, but would benefit greatly from a study that recruited a homogeneous population treated with antipsychotics, and one in which metabolic effects are not obfuscated by exposure to previous medications. A key advantage of conducting the present study at the Vancouver/Richmond EPI program is that it is the only one in British Columbia to have implemented a metabolic screening program, where detailed information is available about the metabolic health of patients from the time that they entered the program. Using genetic information to predict drug responses would allow a greater number of patients to benefit from the use of antipsychotic drugs with personalized assessments of therapeutic efficacy and side effect profiles (69).

#### Hypothesis

Genetic variants will be identified that predict metabolic responses to antipsychotic drugs. The whole exome analysis will detect variations governing metabolic dysregulation in key physiological pathways known *a priori* to regulate metabolism.

#### Protocol

We are conducting a multi-year study to obtain the genomic DNA from approximately 200 patients registered with the Vancouver/Richmond EPI program. Intake is naturalistic, as we recruit all potential patients who are currently being treated with an antipsychotic. All subjects who take part in this study are asked to have their medical charts reviewed for clinical data (especially clinical blood work results), which includes current medications, pre-existing metabolic risks (e.g., smoking, obesity, etc.), height, weight, waist circumference, blood pressure, heart rate, and fasting metabolic indices (plasma glucose, insulin, triglycerides, and cholesterol). General clinical data on demographics, health history, prior and concomitant medications, and any other metabolic indices are also collected from the medical charts (the metabolic indices are the same as in the IHC trial). Any current medical problems experienced in the last 3 days in the form of antipsychotic medication side effects will be captured with the Udvalg for Kliniske Undersøgelser (UKU) Side Effect Rating Scale. Similar to the IHC trial, information on diagnoses in patients participating in this trial will include the clinical diagnoses, and descriptions of positive and negative symptoms, affect abnormalities, problems in social and occupational functioning, and cognitive problems. To accurately phenotype this patient population in relation to antipsychotic-induced metabolic dysfunction, the 48-item UKU is conducted on all subjects to produce a comprehensive measure of the severity and frequency of psychic, neurological, autonomic, and other side effects induced by antipsychotic drugs. This assessment is accomplished by a semistructured interview with each patient during which the scale is gone through point by point and is supplemented by clinical observation and information obtained from the case records or clinical staff. Medication adherence is assessed using the Medication Adherence Rating Scale.

All subjects are then asked to provide a blood sample for whole-exome sequencing. To obtain the genomic DNA samples, subjects have a total of 22 ml (5.5 teaspoons of venous blood) withdrawn from their arm. Samples will be collected by a certified phlebotomist using approved protocols. Venous blood is extracted from the median cubital vein in the cubital fossa of the arm. Samples are collected directly into two PAXGene collection tubes and may be stored for many years in a Biobank. The blood draw is performed at one time point only and takes place wherever the subject receives medical care. Genomic DNA is isolated and purified using the QIAGEN blood mini kit for DNA. Purified genomic DNA is split into aliquots with one aliquot being used for exome library preparation using the Nextera Rapid Capture Exome kit, which requires only 4 Gb of sequence, with greater read depths possible if needed. Enriched exomes are molecularly barcoded such that multiple exomes can be sequenced simultaneously.

High-throughput sequencing is performed at the UBC Pharmaceutical Sciences Sequencing Center (PSSC). Illumina^®^ hiSeq 2500 instrument and a minimum of 20 million sequence reads are captured for each DNA sample using a 2 × 100 paired-end sequencing protocol. We empirically test the read-depth/coverage relationship by sequencing several samples to a 3× greater depth and then undersample the data bioinformatically. Raw data in the form of .bcl files are converted to fastq and the reads mapped using the GATK platform established by the Broad Institute. Samples are tracked by the PSSC’s Laboratory Information Management System by unique identifiers only. Mapped sequences associated with each identifier are stored on the local network for analysis.

#### Significance

Based on the highly detailed metabolic phenotype we will measure, we anticipate that the genomic findings from the current proposal will provide important novel targets to help us understand the metabolic side effects of antipsychotic drugs, either in terms of predicting which drugs may be better or worse for specific individuals, or indicating potential physiological pathways to study for future drug development. We will then hopefully be able to integrate such findings into our active preclinical and clinical programs.

## Concluding Remarks

The beneficial effects of EPI programs on mental health and functional outcome are now well established in patients with a psychotic disorder. However, the long-term physical health of individuals who are treated with antipsychotic medications should remain a priority, given their increased risk for cardiometabolic disorders. The Vancouver/Richmond EPI program has been at the forefront of this challenge. The multidisciplinary team is committed to improving lifelong physical health by a number of important programs that involve working with and educating patients and their families about health issues, while also ensuring regular metabolic monitoring by expert medical staff. This strong focus on improving physical health has created an excellent environment for conducting high quality and “cutting edge” research, whereby patients become more empowered to learn about their own physical health, and also be a part of clinical research that may ultimately improve the physical health of many others.

## Conflict of Interest Statement

Dr. Alasdair M. Barr has received research grant support from Bristol-Myers Squibb. The other co-authors declare that the research was conducted in the absence of any commercial or financial relationships that could be construed as a potential conflict of interest.

## References

[1] Goulding SM Holtzman CW Trotman HD Ryan AT Macdonald AN Shapiro DI The prodrome and clinical risk for psychotic disorders. Child Adolesc Psychiatr Clin N Am (2013) 22:557–67 10.1016/j.chc.2013.04.002 24012073 PMC4140174

[2] Larsen TK McGlashan TH Moe LC First-episode schizophrenia: I. Early course parameters. Schizophr Bull (1996) 22:241–56 10.1093/schbul/22.2.241 8782284

[3] Gerson R Davidson L Booty A McGlashan T Malespina D Pincus HA Families’ experience with seeking treatment for recent-onset psychosis. Psychiatr Serv (2009) 60:812–6 10.1176/appi.ps.60.6.812 19487352 PMC3898847

[4] McGlashan TH Johannessen JO Early detection and intervention with schizophrenia: rationale. Schizophr Bull (1996) 22:201–22 10.1093/schbul/22.2.327 8782282

[5] Nordentoft M Rasmussen JO Melau M Hjorthoj CR Thorup AA How successful are first episode programs? A review of the evidence for specialized assertive early intervention. Curr Opin Psychiatry (2014) 27:167–72 10.1097/YCO.0000000000000052 24662959

[6] Birchwood M Early intervention and sustaining the management of vulnerability. Aust N Z J Psychiatry (2000) 34(Suppl):S181–4 10.1046/j.1440-1614.2000.00791.x 11129305

[7] Marshall M Lewis S Lockwood A Drake R Jones P Croudace T Association between duration of untreated psychosis and outcome in cohorts of first-episode patients: a systematic review. Arch Gen Psychiatry (2005) 62:975–83 10.1001/archpsyc.62.9.975 16143729

[8] Harvey PO Lepage M Malla A Benefits of enriched intervention compared with standard care for patients with recent-onset psychosis: a metaanalytic approach. Can J Psychiatry (2007) 52:464–72 17688011 10.1177/070674370705200709

[9] Bertelsen M Jeppesen P Petersen L Thorup A Ohlenschlaeger J le Quach P Five-year follow-up of a randomized multicenter trial of intensive early intervention vs standard treatment for patients with a first episode of psychotic illness: the OPUS trial. Arch Gen Psychiatry (2008) 65:762–71 10.1001/archpsyc.65.7.762 18606949

[10] Norman RM Manchanda R Malla AK Windell D Harricharan R Northcott S Symptom and functional outcomes for a 5 year early intervention program for psychoses. Schizophr Res (2011) 129:111–5 10.1016/j.schres.2011.04.006 21549566

[11] Raedler TJ Cardiovascular aspects of antipsychotics. Curr Opin Psychiatry (2010) 23:574–81 10.1097/YCO.0b013e32833f46c9 20838345

[12] Casey DA Rodriguez M Northcott C Vickar G Shihabuddin L Schizophrenia: medical illness, mortality, and aging. Int J Psychiatry Med (2011) 41:245–51 10.2190/PM.41.3.c 22073763

[13] Barr AM Procyshyn RM Hui P Johnson JL Honer WG Self-reported motivation to smoke in schizophrenia is related to antipsychotic drug treatment. Schizophr Res (2008) 100:252–60 10.1016/j.schres.2007.11.027 18178062

[14] Laursen TM Munk-Olsen T Vestergaard M Life expectancy and cardiovascular mortality in persons with schizophrenia. Curr Opin Psychiatry (2012) 25:83–8 10.1097/YCO.0b013e32835035ca 22249081

[15] Smith GN Wong H MacEwan GW Kopala LC Ehmann TS Thornton AE Predictors of starting to smoke cigarettes in patients with first episode psychosis. Schizophr Res (2009) 108:258–64 10.1016/j.schres.2008.12.013 19162444

[16] Leung JY Barr AM Procyshyn RM Honer WG Pang CC Cardiovascular side-effects of antipsychotic drugs: the role of the autonomic nervous system. Pharmacol Ther (2012) 135:113–22 10.1016/j.pharmthera.2012.04.003 22565090

[17] Boyda HN Tse L Procyshyn RM Honer WG Barr AM Preclinical models of antipsychotic drug-induced metabolic side effects. Trends Pharmacol Sci (2010) 31:484–97 10.1016/j.tips.2010.07.002 20674990

[18] Procyshyn RM Wasan KM Thornton AE Barr AM Chen EY Pomarol-Clotet E Changes in serum lipids, independent of weight, are associated with changes in symptoms during long-term clozapine treatment. J Psychiatry Neurosci (2007) 32:331–8 17823649 PMC1963353

[19] Leung JY Pang CC Procyshyn RM Barr AM Cardiovascular effects of acute treatment with the antipsychotic drug olanzapine in rats. Vascul Pharmacol (2014). 10.1016/j.vph.2014.06.003 24969105

[20] Procyshyn RM Su J Elbe D Liu AY Panenka WJ Davidson J Prevalence and patterns of antipsychotic use in youth at the time of admission and discharge from an inpatient psychiatric facility. J Clin Psychopharmacol (2014) 34:17–22 10.1097/JCP.0b013e3182a607dd 24346744

[21] Linton D Barr AM Honer WG Procyshyn RM Antipsychotic and psychostimulant drug combination therapy in attention deficit/hyperactivity and disruptive behavior disorders: a systematic review of efficacy and tolerability. Curr Psychiatry Rep (2013) 15:355 10.1007/s11920-013-0355-6 23539465

[22] Procyshyn RM Honer WG Wu TK Ko RW McIsaac SA Young AH Persistent antipsychotic polypharmacy and excessive dosing in the community psychiatric treatment setting: a review of medication profiles in 435 Canadian outpatients. J Clin Psychiatry (2010) 71:566–73 10.4088/JCP.08m04912gre 20361903

[23] Honer WG Thornton AE Sherwood M MacEwan GW Ehmann TS Williams R Conceptual and methodological issues in the design of clinical trials of antipsychotics for the treatment of schizophrenia. CNS Drugs (2007) 21:699–714 10.2165/00023210-200721090-00001 17696571

[24] American Diabetes Association; American Psychiatric Association; American Association of Clinical Endocrinologists; North American Association for the Study of Obesity. Consensus development conference on antipsychotic drugs and obesity and diabetes. Diabetes Care (2004) 27:596–601 10.2337/diacare.27.2.596 14747245

[25] Grundy SM Brewer HB Jr Cleeman JI Smith SC Jr Lenfant C Definition of metabolic syndrome: report of the National Heart, Lung, and Blood Institute/American Heart Association conference on scientific issues related to definition. Arterioscler Thromb Vasc Biol (2004) 24:e13–8 10.1161/01.ATV.0000111245.75752.C6 14766739

[26] American Heart Association. Metabolic syndrome: new guidance for prevention and treatment. AHA & NHLBI Scientific Statement (2005).

[27] Manu P Correll CU van Winkel R Wampers M De Hert M Prediabetes in patients treated with antipsychotic drugs. J Clin Psychiatry (2012) 73:460–6 10.4088/JCP.10m06822 22225552

[28] Meyer J Loh C Leckband SG Boyd JA Wirshing WC Pierre JM Prevalence of the metabolic syndrome in veterans with schizophrenia. J Psychiatr Pract (2006) 12:5–10 10.1097/00131746-200601000-00002 16432440

[29] Malhi G Adams D Plain J Coulston C Herman M Walter G Clozapine and cardiometabolic health in chronic schizophrenia: correlations and consequences in a clinical context. Australas Psychiatry (2010) 18:32–41 10.3109/10398560903254193 20039791

[30] McEvoy JP Meyer JM Goff DC Nasrallah HA Davis SM Sullivan L Prevalence of the metabolic syndrome in patients with schizophrenia: baseline results from the Clinical Antipsychotic Trials of Intervention Effectiveness (CATIE) schizophrenia trial and comparison with national estimates from NHANES III. Schizophr Res (2005) 80:19–32 10.1016/j.schres.2005.07.014 16137860

[31] Correll CU Carlson HE Endocrine and metabolic adverse effects of psychotropic medications in children and adolescents. J Am Acad Child Adolesc Psychiatry (2006) 45:771–91 10.1097/01.chi.0000220851.94392.30 16832314

[32] Martinez-Ortega JM Funes-Godoy S Diaz-Atienza F Gutierrez-Rojas L Perez-Costillas L Gurpegui M Weight gain and increase of body mass index among children and adolescents treated with antipsychotics: a critical review. Eur Child Adolesc Psychiatry (2013) 22:457–79 10.1007/s00787-013-0399-5 23503976

[33] Maayan L Correll CU Weight gain and metabolic risks associated with antipsychotic medications in children and adolescents. J Child Adolesc Psychopharmacol (2011) 21:517–35 10.1089/cap.2011.0015 22166172

[34] Bovend’Eerdt TJ Botell RE Wade DT Writing SMART rehabilitation goals and achieving goal attainment scaling: a practical guide. Clin Rehabil (2009) 23:352–61 10.1177/0269215508101741 19237435

[35] Marshall M Rathbone J Early intervention for psychosis. Cochrane Database Syst Rev (2006) (4):CD004718 10.1002/14651858.CD004718.pub2 17054213

[36] Mangurian C Giwa F Shumway M Fuentes-Afflick E Perez-Stable EJ Dilley JW Primary care providers’ views on metabolic monitoring of outpatients taking antipsychotic medication. Psychiatr Serv (2013) 64:597–9 10.1176/appi.ps.002542012 23728604 PMC3780562

[37] Heald A Montejo AL Millar H De Hert M McCrae J Correll CU Management of physical health in patients with schizophrenia: practical recommendations. Eur Psychiatry (2010) 25(Suppl 2):S41–5 10.1016/S0924-9338(10)71706-5 20620887

[38] Tse L Procyshyn RM Fredrikson DH Boyda HN Honer WG Barr AM Pharmacological treatment of antipsychotic-induced dyslipidemia and hypertension. Int Clin Psychopharmacol (2014) 29:125–37 10.1097/YIC.0000000000000014 24169026

[39] Lang DJ Barr AM Procyshyn RM Management of medication-related cardiometabolic risk in patients with severe mental illness. Curr Cardiovasc Risk Rep (2013) 7:283–7 10.1007/s12170-013-0321-1 23864926 PMC3702958

[40] Reynolds GP Kirk SL Metabolic side effects of antipsychotic drug treatment – pharmacological mechanisms. Pharmacol Ther (2010) 125:169–79 10.1016/j.pharmthera.2009.10.010 19931306

[41] Boyda HN Procyshyn RM Pang CC Hawkes E Wong D Jin CH Metabolic side-effects of the novel second-generation antipsychotic drugs asenapine and iloperidone: a comparison with olanzapine. PLoS One (2013) 8:e53459 10.1371/journal.pone.0053459 23326434 PMC3541274

[42] Boyda HN Procyshyn RM Tse L Xu J Jin CH Wong D Antipsychotic polypharmacy increases metabolic dysregulation in female rats. Exp Clin Psychopharmacol (2013) 21:164–71 10.1037/a0031228 23356730

[43] Barr AM Wu CH Wong C Hercher C Topfer E Boyda HN Effects of chronic exercise and treatment with the antipsychotic drug olanzapine on hippocampal volume in adult female rats. Neuroscience (2013) 255:147–57 10.1016/j.neuroscience.2013.10.010 24141179

[44] Boyda HN Procyshyn RM Tse L Wong D Pang CC Honer WG Intermittent treatment with olanzapine causes sensitization of the metabolic side-effects in rats. Neuropharmacology (2012) 62:1391–400 10.1016/j.neuropharm.2011.02.019 21376062

[45] Boyda HN Tse L Procyshyn RM Wong D Wu TK Pang CC A parametric study of the acute effects of antipsychotic drugs on glucose sensitivity in an animal model. Prog Neuropsychopharmacol Biol Psychiatry (2010) 34:945–54 10.1016/j.pnpbp.2010.04.024 20452386

[46] Boyda HN Procyshyn RM Asiri Y Wu C Wang CK Lo R Antidiabetic-drug combination treatment for glucose intolerance in adult female rats treated acutely with olanzapine. Prog Neuropsychopharmacol Biol Psychiatry (2014) 48:170–6 10.1016/j.pnpbp.2013.10.006 24140931

[47] Boyda HN Ramos-Miguel A Procyshyn RM Topfer E Lant N Choy HH Routine exercise ameliorates the metabolic side-effects of treatment with the atypical antipsychotic drug olanzapine in rats. Int J Neuropsychopharmacol (2014) 17:77–90 10.1017/S1461145713000795 23953063

[48] Boyda HN Procyshyn RM Tse L Hawkes E Jin CH Pang CC Differential effects of 3 classes of antidiabetic drugs on olanzapine-induced glucose dysregulation and insulin resistance in female rats. J Psychiatry Neurosci (2012) 37:407–15 10.1503/jpn.110140 22640703 PMC3493097

[49] Edelman D Oddone EZ Liebowitz RS Yancy WS Jr Olsen MK Jeffreys AS A multidimensional integrative medicine intervention to improve cardiovascular risk. J Gen Intern Med (2006) 21:728–34 10.1111/j.1525-1497.2006.00495.x 16808774 PMC1924710

[50] Yang NY Wroth S Parham C Strait M Simmons LA Personalized health planning with integrative health coaching to reduce obesity risk among women gaining excess weight during pregnancy. Glob Adv Health Med (2013) 2:72–7 10.7453/gahmj.2013.033 24278848 PMC3833555

[51] Moore C Case report of hemoglobin a1c and weight reduction in integrative health coaching. Glob Adv Health Med (2013) 2:87–9 10.7453/gahmj.2013.016 24278844 PMC3833540

[52] Wolever RQ Dreusicke M Fikkan J Hawkins TV Yeung S Wakefield J Integrative health coaching for patients with type 2 diabetes: a randomized clinical trial. Diabetes Educ (2010) 36:629–39 10.1177/0145721710371523 20534872

[53] Naik AD White CD Robertson SM Armento ME Lawrence B Stelljes LA Behavioral health coaching for rural-living older adults with diabetes and depression: an open pilot of the HOPE study. BMC Geriatr (2012) 12:37 10.1186/1471-2318-12-37 22828177 PMC3542105

[54] Heggelund J Morken G Helgerud J Nilsberg GE Hoff J Therapeutic effects of maximal strength training on walking efficiency in patients with schizophrenia – a pilot study. BMC Res Notes (2012) 5:344 10.1186/1756-0500-5-344 22759719 PMC3568714

[55] Blouin M Tremblay A Jalbert ME Venables H Bouchard RH Roy MA Adiposity and eating behaviors in patients under second generation antipsychotics. Obesity (Silver Spring) (2008) 16:1780–7 10.1038/oby.2008.277 18535555

[56] Barak Y Aizenberg D Switching to aripiprazole as a strategy for weight reduction: a meta-analysis in patients suffering from schizophrenia. J Obes (2011) 2011 10.1155/2011/898013 20871835 PMC2943136

[57] Tandon R Halbreich U The second-generation ‘atypical’ antipsychotics: similar improved efficacy but different neuroendocrine side effects. Psychoneuroendocrinology (2003) 28(Suppl 1):1–7 10.1016/S0306-4530(02)00109-9 12504068

[58] Kuk JL Katzmarzyk PT Nichaman MZ Church TS Blair SN Ross R Visceral fat is an independent predictor of all-cause mortality in men. Obesity (Silver Spring) (2006) 14:336–41 10.1038/oby.2006.43 16571861

[59] Demerath EW Reed D Rogers N Sun SS Lee M Choh AC Visceral adiposity and its anatomical distribution as predictors of the metabolic syndrome and cardiometabolic risk factor levels. Am J Clin Nutr (2008) 88:1263–71 18996861 10.3945/ajcn.2008.26546 PMC2801427

[60] Druet C Baltakse V Chevenne D Dorgeret S Zaccaria I Wang Y Independent effect of visceral adipose tissue on metabolic syndrome in obese adolescents. Horm Res (2008) 70:22–8 10.1159/000129674 18493146

[61] Usui C Asaka M Kawano H Aoyama T Ishijima T Sakamoto S Visceral fat is a strong predictor of insulin resistance regardless of cardiorespiratory fitness in non-diabetic people. J Nutr Sci Vitaminol (Tokyo) (2010) 56:109–16 10.3177/jnsv.56.109 20495292

[62] Bosy-Westphal A Booke CA Blocker T Kossel E Goele K Later W Measurement site for waist circumference affects its accuracy as an index of visceral and abdominal subcutaneous fat in a Caucasian population. J Nutr (2010) 140:954–61 10.3945/jn.109.118737 20335625

[63] Hwang JH Stein DT Barzilai N Cui MH Tonelli J Kishore P Increased intrahepatic triglyceride is associated with peripheral insulin resistance: in vivo MR imaging and spectroscopy studies. Am J Physiol Endocrinol Metab (2007) 293:E1663–9 10.1152/ajpendo.00590.2006 17911339

[64] Korenblat KM Fabbrini E Mohammed BS Klein S Liver, muscle, and adipose tissue insulin action is directly related to intrahepatic triglyceride content in obese subjects. Gastroenterology (2008) 134:1369–75 10.1053/j.gastro.2008.01.075 18355813 PMC2629391

[65] Machann J Thamer C Stefan N Schwenzer NF Kantartzis K Haring HU Follow-up whole-body assessment of adipose tissue compartments during a lifestyle intervention in a large cohort at increased risk for type 2 diabetes. Radiology (2010) 257:353–63 10.1148/radiol.10092284 20713612

[66] Zhang ZJ Yao ZJ Liu W Fang Q Reynolds GP Effects of antipsychotics on fat deposition and changes in leptin and insulin levels. Magnetic resonance imaging study of previously untreated people with schizophrenia. Br J Psychiatry (2004) 184:58–62 10.1192/bjp.184.1.58 14702228

[67] Adkins DE Aberg K McClay JL Bukszar J Zhao Z Jia P Genomewide pharmacogenomic study of metabolic side effects to antipsychotic drugs. Mol Psychiatry (2011) 16:321–32 10.1038/mp.2010.14 20195266 PMC2891163

[68] Zhang JP Malhotra AK Pharmacogenetics and antipsychotics: therapeutic efficacy and side effects prediction. Expert Opin Drug Metab Toxicol (2011) 7:9–37 10.1517/17425255.2011.532787 21162693 PMC3057913

[69] Zhang JP Malhotra AK Pharmacogenetics of antipsychotics: recent progress and methodological issues. Expert Opin Drug Metab Toxicol (2013) 9:183–91 10.1517/17425255.2013.736964 23199282 PMC3547146

